# Pulmonary vein thrombosis associated with metastatic uterine leiomyosarcoma: a case report

**DOI:** 10.1186/s13256-022-03719-7

**Published:** 2023-01-12

**Authors:** Mehdi Salimi, Dena Mohamadzadeh, Mojdeh Bonyadi

**Affiliations:** Clinical Research Development Center, Imam Reza Hospital, Kermanshah University of Medical Sciences, Kermanshah, Iran

**Keywords:** Case report, Leiomyosarcoma, Malignancy, Pulmonary vein thrombosis

## Abstract

**Background:**

Pulmonary vein thrombosis (PVT) is rarely associated with malignancies. Leiomyosarcoma, a malignant tumor originating from smooth muscles, has never been reported as the etiology of PVT.

**Case presentation:**

In this case report, we described a 43-year-old Kurdish woman with a known case of leiomyosarcoma who presented with hemoptysis, dyspnea, and pleuritic chest pain. Chest computed tomography (CT) angiography revealed a thrombus in the left infero-posterior pulmonary vein. She was successfully treated with unfractionated heparin administered intravenously followed by orally administered warfarin. At the end of the article, we describe and compare other reports of malignancy-related PVT.

**Conclusions:**

While malignancies are not a common cause of PVT, both primary lung tumors and metastatic cancers could be associated with PVT. Delay in diagnosis may lead to serious complications and even death. Therefore, clinicians should be aware of the possibility of the development of PVT in different malignancies for appropriate diagnosis and treatment.

## Background

Pulmonary vein thrombosis (PVT) is a rare condition. Different etiologies have been defined for this rare disease. Lung transplantation is the most common condition associated with PVT. Another rare etiology for PVT is malignancies [1]. Uterine leiomyosarcoma is a rare malignant tumor that originates from smooth muscle cells. It mostly spreads hematogenously. While metastasis to the lungs is a well-recognized complication of this tumor [2], as far as we know, no previous article has reported PVT associated with leiomyosarcoma.

Here, we report the first case of PVT associated with metastatic uterine leiomyosarcoma, in efforts to raise awareness of this important but treatable complication.

## Case presentation

The patient was a 43-year-old Kurdish woman who presented to the emergency department of our hospital with a 2-day history of hemoptysis, dyspnea, and pleuritic chest pain. She also complained of a 10-day history of overnight fever and chills, and generalized arthralgia 20 days prior to the recent admission. Her past medical history was unremarkable except for uterine leiomyosarcoma which was diagnosed 5 months earlier. Abdominopelvic computed tomography (CT) scan showed a large solid mass measuring 113 mm × 206 mm which was located superior to the uterus and was connected by a stalk to the uterine fundus. A core needle biopsy confirmed the diagnosis. She underwent a hysterectomy and left salpingo-oophorectomy 1 month prior to the recent hospitalization. Chest CT scan was performed before surgery and did not show metastatic lesions. She did not use any drugs prior to admission and had not received chemotherapy for sarcoma. Her familial history was unremarkable for malignancies or thrombotic events. She denied alcohol or cigarette consumption. She was single, a housekeeper, and had no children.

On the day of admission, her vital signs were as follows: heart rate = 120 beats/minute, respiratory rate = 20/minute, blood pressure = 125/80 mmHg, temperature = 37 °C. Oxygen saturation was 95% on room air. On general examination, she was neither pale nor cyanotic. Respiratory distress was not detected. Results of physical examination of the respiratory and cardiovascular systems were within normal limits. Lung auscultation and percussion were normal. Examination of the limbs was unremarkable except for 1+ pitting edema of both lower extremities up to the ankles. No arthritis was obvious, and the range of motion of the joints was normal. Examination of cranial nerves was unremarkable. Deep tendon reflexes were 2+ and bilateral plantar reflexes were downward. Proximal and distal muscle strength was 5/5.

Laboratory test results on the day of admission were as follows: hemoglobin of 12.8 mg/dL, mean corpuscular volume (MCV) 84 fl, white blood cell count (WBC) 10.2 × 10^3^/mm^3^ (differential count: neutrophils 78%, lymphocytes 12%, monocytes 10%), platelet count 363 × 10^3^/mm^3^, creatinine 0.7 mg/dL, international normalized ratio (INR) 1, prothrombin time (PTT) 24 seconds, lactate dehydrogenase (LDH) 290 IU/mL (normal: 207–414 IU/mL), aspartate transaminase (AST) 31 IU/L (normal: 0–31 IU/L), alanine transaminase (ALT) 27 IU/L (normal: 0–34 IU/L), alkaline phosphatase (ALP) 213 U/L (normal: 64–306 U/L), erythrocyte sedimentation rate (ESR) 119 mm/hour, and C-reactive protein (CRP) 2+. Venous blood gas (VBG) was as follows: pH = 7.52, partial pressure of carbon dioxide (PCO2) = 34, bicarbonate (HCO3) = 28.

**Fig. 1:**
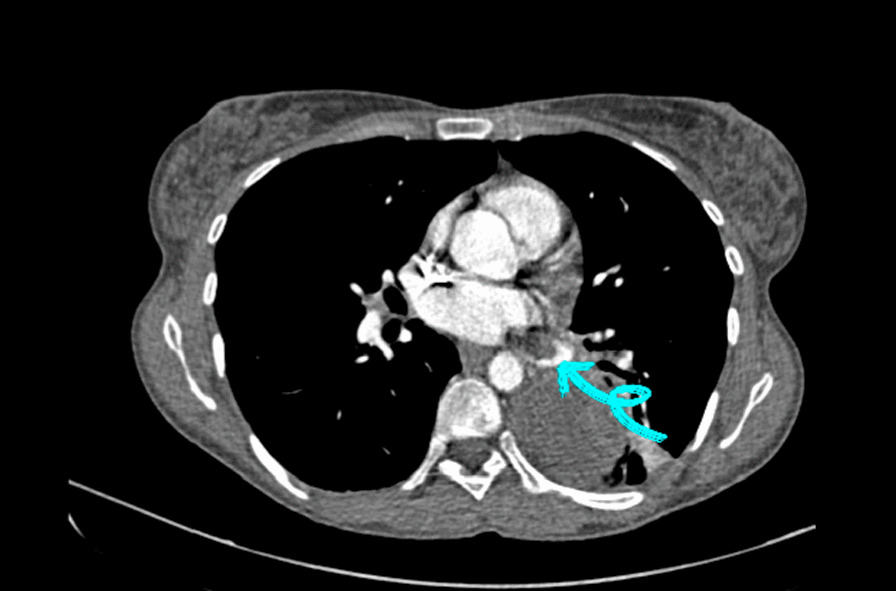
Mediastinal view of the chest computed tomography angiography: blue arrow shows the thrombus in the left infero-posterior pulmonary vein

The calculated score for the Wells criteria was 6.5 (score of 1 for hemoptysis, 1 for having cancer, 1.5 for tachycardia, and 3 for having no alternative diagnosis more likely than pulmonary thromboembolism [PTE]) [3]. As a result, PTE was suspected, and CT pulmonary angiography was performed. Multiple soft tissue nodules were seen in the field of both lungs suggestive of metastatic lesions. A mass measuring 54 mm × 46 mm with density similar to soft tissue was detected in the apicoposterior segment of the left lower lobe. A thrombus was observed in the left infero-posterior pulmonary vein (Fig. 1). Transthoracic echocardiography recorded a left ventricular ejection fraction of 55% and pulmonary artery pressure of 35 mmHg. The right ventricle size was normal. She was started on unfractionated heparin 80 units/kg immediately and 8 units/kg per hour intravenously. Administration of warfarin tablets 5 mg/day was started the next day. Five days after reaching INR > 2, heparin was discontinued and she was discharged in stable condition with warfarin tablets 5 mg/day. After discharge, she was referred to a radiation oncologist for the management of metastatic cancer. She was followed up in the pulmonology clinic about 5 months later. She was on warfarin 5 mg daily and she had no recurrence of symptoms. She was receiving gemcitabine 675 mg/m^2^ every 21 days intravenously in combination with 100 mg/m^2^ docetaxel on day 8 intravenously as a chemotherapy regimen for metastatic uterine leiomyosarcoma.

## Discussion and conclusions

The patient was a 43-year-old woman with metastatic leiomyosarcoma, complaining of hemoptysis, dyspnea, and pleuritic chest pain. PVT was confirmed by chest CT angiography. She was treated with anticoagulants. As far as we know, no previous article has reported metastatic leiomyosarcoma as a cause of PVT. PVT is a relatively rare condition, but it can be life-threatening and lead to serious complications. Different etiologies have been identified for PVT including lung transplantation and lobectomy, atrial fibrillation, malignancies, and idiopathic etiology. Surgical procedures affecting pulmonary veins such as lung transplantation are the most frequently reported cause of PVT. Malignancies are another known but rare cause. Primary lung cancers are the most common malignancies associated with PVT [1]. Leivaditis *et al*. [4] reported giant cell lung carcinoma and Faiek *et al*. [5] reported bronchogenic carcinoma causing PVT. However, metastatic cancers such as osteogenic carcinoma [6], intestinal mantle cell lymphoma [7], liposarcoma [8], choriocarcinoma [9], follicular thyroid carcinoma [10], polycythemia vera [11], and renal cell carcinoma [12] have also been reportedly associated with PVT. In this article, we report PVT in a 43-year old woman with uterine leiomyosarcoma.

Clinical presentation varies from asymptomatic cases to nonspecific symptoms which may mimic pulmonary arterial embolism, such as hemoptysis, dyspnea, and cough. Our patient presented with dyspnea, pleuritic chest pain, and hemoptysis. PTE was initially suspected. The presentation may be more insidious, such as recurrent pulmonary edema and lung fibrosis [7]. Diagnosis of PVT may be difficult due to the nonspecific sign and symptoms. Different diagnostic modalities include pulmonary CT angiography, magnetic resonance imaging (MRI) of the chest, and transesophageal echocardiography (TEE) [1]. In our patient, the diagnosis was made by pulmonary CT angiography. The thrombus was located at the branches of both the right [5–7, 9, 10, 12] and left [4, 8, 11] pulmonary veins. In our patient, thrombosis was observed in the left infero-posterior pulmonary vein.

Different treatments have been reported for PVT including antibiotic therapy, anticoagulation, surgical resection of the thrombus, and pulmonary resection [1]. Our patient was hemodynamically stable and was successfully treated with unfractionated heparin; she was discharged with warfarin 5 mg daily. Nelson *et al*. [6] and Leivaditis *et al*. [4] reported successful treatment of malignancy-related PVT by surgical thrombus resection. The other previously reported cases were treated by anticoagulant therapy [5, 7–12]. Of these, two patients [7, 8], a 40-year-old woman with liposarcoma and a 66-year-old man with mantle cell sarcoma of the intestine, died.

We investigated ten cases of PVT associated with different malignancies (nine are the previously reported cases and the last one is the present case) [4–12]. The mean age of the patients was 58.1 years (the youngest was a 29-year-old woman with osteogenic carcinoma, and the oldest was a 76-year-old man with polycythemia vera). Half of the patients were female, and the other half were male. In two cases, PVT was a complication of primary lung cancer (giant cell lung carcinoma [4] and bronchogenic carcinoma [5]). The remaining patients had metastatic cancers with different origins. In six cases [5–7, 9, 10, 12], the thrombus was located at the branches of the right pulmonary vein. In the other four cases, including the present case, it was located at the branches of the left pulmonary vein [4, 8, 11]. Two of the patients (a 29-year-old woman with osteogenic carcinoma [6] and a 61-year-old man with giant cell lung carcinoma [4]) were successfully treated with surgical resection of the thrombus. The other four patients were started on anticoagulant therapy, and two of them [7, 8] succumbed to the illness. Table 1 includes information on these ten cases of PVT associated with different malignancies.

**Table 1:** Articles reporting pulmonary vein thrombus associated with malignant tumors

Articles	Age (years)/sex	Type of tumor	Site of thrombus	Treatment	Outcome
Nelson *et al*. [6]	29/F	Osteogenic carcinoma	Right inferior pulmonary vein	Thrombus resection	Improved
Leivaditis *et al*. [4]	61/M	Giant cell lung carcinoma	Upper left pulmonary vein	Thrombus resection	Improved
Akiode *et al*. [7]	66/M	Small cell lung carcinoma/intestinal mantle cell lymphoma	Inferior branch of right pulmonary vein	Anticoagulant	Died
Faiek *et al*. [5]	67/M	Bronchogenic carcinoma	Right inferior pulmonary vein	Anticoagulant	Improved
Tamizifar *et al*. [8]	40/F	Liposarcoma	Left inferior pulmonary vein	Anticoagulant	Died
Bonnet *et al*. [9]	57/F	Choriocarcinoma	Right inferior pulmonary vein	Anticoagulant	Improved
Mavromati *et al*. [10]	69/F	Follicular thyroid carcinoma	Right superior pulmonary vein	Anticoagulant	Improved
Bhardwaj *et al*. [11]	76/M	Polycythemia vera	Left inferior pulmonary vein	Anticoagulant	Improved
Stein *et al*. [12]	73/M	Renal cell carcinoma	Right superior pulmonary vein	–	–
Salimi *et al*. (present case)	43/F	Leiomyosarcoma of uterine	Left infero-posterior pulmonary vein	Anticoagulant	Improved

*F* female, *M* male

We conclude that pulmonary vein thrombus can be associated with primary lung malignancies or metastatic cancers. Branches of both left and right pulmonary veins can be involved. Improvement can be achieved by anticoagulant therapy or surgical resection of the thrombus. Our effort through this article was to raise awareness of this rare but important condition, as delayed diagnosis leads to serious complications and even death.

## Data Availability

Available if requested.

## Abbreviations

PVT: Pulmonary vein thrombosis
PTE: Pulmonary thromboembolism
CT: Computed tomography
TEE: Transesophageal echocardiography
